# EchoBack-CAR T cells: a sonogenetic solution to the persistent challenges of solid tumor immunotherapy

**DOI:** 10.1038/s41392-025-02387-5

**Published:** 2025-09-18

**Authors:** Kiseok Han, Jung-Soo Suh, Tae-Jin Kim

**Affiliations:** 1 Department of Integrated Biological Science, Pusan National University, Busan, Republic of Korea; 2 Department of Biological Sciences, Pusan National University, Busan, Republic of Korea; 3 Institute of Systems Biology, Pusan National University, Busan, Republic of Korea

**Keywords:** Immunotherapy, Molecular engineering

**Fig. 1:**
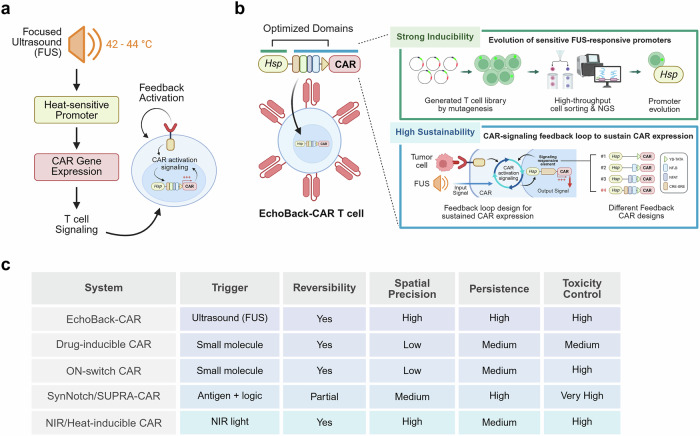
**a** Schematic of the EchoBack-CAR T cell system. Focused ultrasound (FUS)-induced local hyperthermia activates a heat-sensitive promoter to initiate CAR expression, which in turn triggers antigen-dependent T cell signaling. The resulting intracellular signaling enhances promoter activity, forming a positive feedback loop that sustains CAR expression. **b** Schematic showing the engineering strategy for optimizing the EchoBack-CAR gene circuit. A library of heat-responsive promoters was screened to identify constructs with minimal basal activity and strong inducibility. To enable prolonged CAR expression following transient FUS stimulation, enhancer elements responsive to CAR signaling pathways (such as NFAT and NF-κB) were incorporated, establishing a self-amplifying gene circuit. **c** Comparison of different optimized CAR-T systems. This figure was created with BioRender.com

In a recent study published in *Cell*, Liu et al.^1^ introduced EchoBack-CAR, a sonogenetic chimeric antigen receptor T cell platform that enables remote, spatiotemporal activation of CAR expression using focused ultrasound (FUS). By combining an evolved heat-inducible promoter with a synthetic positive feedback loop, EchoBack-CAR achieves potent antitumor effects while minimizing off-tumor toxicity. This system offers a promising solution for treating solid tumors, which have remained a significant challenge for conventional CAR-T therapies (Fig. 1a, b).

Although CAR T therapy has demonstrated long-term success in treating hematologic malignancies such as B cell leukemias and lymphomas, these results have not translated effectively to solid tumors.^2^ Key obstacles include antigen heterogeneity, immunosuppressive microenvironments, and on-target, off-tumor (OTOT) toxicity. Efforts to address these limitations have led to the development of tumor microenvironment-sensing logic gates, transient mRNA CARs, and synthetic switches. However, many of these approaches struggle to balance antitumor efficacy with systemic safety.

Recently, a variety of CAR-T control platforms have emerged, offering different combinations of spatial precision, reversibility, persistence, and toxicity control (Fig. 1c). Drug-inducible and ON-switch CARs allow reversible activation using small molecules but lack spatial resolution. SynNotch and SUPRA-CAR systems improve toxicity control through antigen-dependent logic gating but offer only moderate persistence and spatial precision. In contrast, EchoBack-CAR and NIR/heat-inducible CARs utilize external triggers to achieve high spatial precision and reversibility, though their persistence and toxicity control differ. EchoBack-CAR offers a balanced profile by integrating ultrasound responsiveness with engineered feedback regulation.

A central innovation of EchoBack-CAR lies in its heat-inducible promoter. Liu et al. constructed a large heat shock element (HSE) library (16,384 variants) and performed high-throughput screening using Sort-seq. They identified an optimized variant (clone #4), which, when multimerized and fused to a minimal YB-TATA promoter, displayed strong inducibility with minimal basal activity under physiological conditions—even in the presence of cytokines. This is critical, as even low-level CAR expression in off-target tissues can lead to severe toxicity, as shown in prior clinical studies involving high-affinity HER2 or GD2 CARs.^3^

To extend CAR expression beyond the transient effect of heat shock, the authors incorporated a synthetic positive feedback loop. This design reprograms transient CAR signaling into sustained expression by embedding transcription factor binding sites (responsive to NF-κB, NFAT, cAMP, and MAPK pathways) upstream of the promoter. Upon antigen engagement, these intracellular signals further activate the promoter, reinforcing CAR expression and maintaining T cell function within the tumor microenvironment. This effectively addresses the common issue of functional decay in transient CAR systems.

Functionally, EchoBack-CAR T cells exhibited potent and durable antitumor activity in multiple preclinical models. In 3D glioblastoma spheroid assays, EchoBack-hGD2CAR T cells eradicated tumor cells following two short heat-shock treatments, whereas control inducible CARs allowed tumor regrowth. In vivo, localized FUS activation of EchoBack-CAR T cells suppressed tumor growth in both subcutaneous and orthotopic glioblastoma models and extended mouse survival without apparent toxicity to major organs. Notably, EchoBack-CARs also showed robust efficacy when re-engineered to target prostate-specific membrane antigen (PSMA), a clinically relevant but high-risk antigen expressed in both prostate tumors and healthy kidney and intestinal tissues. Whereas constitutive PSMA-CAR T cells caused cytotoxicity against PSMA-low bystander cells, EchoBack-PSMACAR T cells induced tumor regression while sparing normal tissues—a clear demonstration of spatial control in mitigating OTOT effects.

A particularly insightful aspect of the study is the transcriptomic profiling of CAR T cell states under chronic antigen stimulation. Using single-cell RNA sequencing, the authors found that EchoBack-CAR T cells were enriched in CD8⁺ memory and proliferative effector populations, expressed high levels of cytotoxic mediators such as GZMA and GNLY, and downregulated exhaustion markers including PDCD1, LAG3, and TIGIT. These features were validated by flow cytometry and functional assays, showing that EchoBack-CAR T cells retained proliferative capacity and cytotoxicity after multiple tumor re-challenges, in contrast to conventional CAR T cells, which exhibited a rapid decline in function. These findings support the notion that periodic rest between activations—enabled here by the decay of CAR expression between FUS stimulations—helps prevent T cell exhaustion, an idea supported by previous studies exploring the role of TET2 or transient CAR suppression in rescuing exhausted phenotypes.^4,5^

What distinguishes EchoBack-CAR T cells from other control systems is their ability to combine external, non-invasive, and spatially confined activation with endogenous feedback to sustain therapeutic efficacy. This dual mechanism overcomes the major limitations of both constitutive and transient CAR expression. While other systems have achieved logic-gated or drug-inducible control, they often require continuous administration of small molecules or lack spatial resolution. Focused ultrasound, in contrast, offers millimeter precision and compatibility with clinical imaging modalities, enabling real-time control over CAR T cell activity. Moreover, the modular nature of the EchoBack system makes it readily adaptable to other tumor antigens or signaling circuits.

Nonetheless, several challenges remain before clinical translation. Current efficacy relies on locoregional delivery of CAR T cells and direct ultrasound application to accessible tumors. Achieving systemic trafficking to deep-seated or metastatic lesions will require additional engineering, such as the incorporation of homing receptors or in situ antigen priming strategies. Moreover, the safety of ultrasound activation in antigen-expressing normal tissues must be carefully evaluated in immunocompetent models. Emerging FUS technologies, such as MR-guided adaptive focusing, thermal dosimetry-based feedback, and real-time closed-loop control using motion tracking or acoustic emissions, are rapidly advancing toward clinical translation, enabling precise and safe spatiotemporal regulation of cellular therapies. However, given the expanding toolbox of FUS technologies and CAR design elements, the prospects for refining this approach are strong.

In summary, EchoBack-CAR T cells represent a compelling advance in the design of controllable and persistent cell therapies. By integrating an evolved inducible promoter with a self-amplifying feedback loop, and linking both to a physical activation modality, this platform offers a powerful combination of safety, specificity, and sustained function. As the field moves toward more precise and personalized immunotherapies, EchoBack stands out as a versatile and clinically relevant solution for tackling the unique challenges of solid tumors.
